# Therapeutic Efficacy and Safety of Botulinum Toxin Type A in Erythematotelangiectatic Rosacea: A Systematic Review

**DOI:** 10.7759/cureus.106841

**Published:** 2026-04-11

**Authors:** Khadija Marrar, Rawan Althiyabi, Maria Alsannaa, Renad Alfalah, Jumanah Soroje, Eyad Anbar, Ibtiahl Al Ibrahim, Ameen Al Awadhi

**Affiliations:** 1 College of Medicine, Almarefa University, Riyadh, SAU; 2 College of Health Science and Rehabilitation, Princess Nourah University, Riyadh, SAU; 3 College of Medicine, Royal College of Surgeons in Ireland, Medical University of Bahrain, Busiteen, BHR; 4 Department of Dermatology, King Saud Medical City, Riyadh, SAU; 5 College of Medicine, Umm-Al Qura University, Makkah, SAU; 6 College of Medicine, King Abdulaziz University, Jeddah, SAU; 7 College of Medicine, Imam Abdulrahman Bin Faisal University, Dammam, SAU; 8 Department of Dermatology, Sheikh Khalifa Medical City, Abu Dhabi, ARE

**Keywords:** bont-a, botulinum toxin, botulinum toxin-a (bont-a), etr, rosacea, systematic review, type 1 rosacea

## Abstract

Botulinum toxin type A (BoNT-A) injections have a well-known cosmetic outcome and safety profile in a variety of medical conditions. Recent studies have shown promising results for possible use in treating erythematotelangiectatic rosacea (ETR). This systematic review aimed to evaluate the efficacy and safety of BoNT-A injections in patients with ETR.

A systematic search was conducted across multiple databases to identify relevant studies. Eligible articles were randomized controlled trials (RCTs) that investigated BoNT-A injections in adults diagnosed with ETR. Studies involving other rosacea subtypes, lacking a comparative group, or combining BoNT-A with other interventions without isolating its effects were excluded.

Three RCTs met the inclusion criteria. Both parallel-group and split-face analyses demonstrated that BoNT-A injections significantly reduced facial erythema and flushing, resulting in improved patient satisfaction. Across all studies, BoNT-A was well-tolerated, with adverse events being mild, transient, and self-limited.

However, the evidence remains limited by the small sample sizes, suboptimal study designs, and short follow-up durations. BoNT-A injections show promising efficacy and safety in the treatment of ETR. However, larger, well-designed randomized trials with longer follow-up durations are required to confirm its therapeutic role and establish standardized treatment protocols.

## Introduction and background

Rosacea is a long-standing skin condition that affects many individuals and often interferes with daily life [1]. It is a chronic inflammatory disorder in which patients experience repeated episodes of facial redness. Four major clinical variants are recognized, of which erythematotelangiectatic rosacea (ETR) is the most frequent [1]. This form is characterized by persistent erythema of the face and enlargement of superficial cutaneous blood vessels. Patients may also report sudden flushing together with burning, tingling, or itching, and in some cases, dryness or sensitivity. A variety of triggers, including environmental exposure, dietary factors, stress, or topical irritants, have been linked to these symptoms. In addition, disturbances in neurovascular regulation may contribute to immune imbalance, which is thought to play a role in the disease process. The disorder is most commonly diagnosed between the ages of 30 and 50, with a higher prevalence in women but a greater severity in men [1].

Management of rosacea is guided by expert recommendations and available clinical guidelines, which suggest oral therapies such as doxycycline; topical medications including oxymetazoline, brimonidine, azelaic acid, and ivermectin; and the use of light or laser-based treatments to reduce erythema and telangiectasia [2]. Nevertheless, treating rosacea can be difficult, particularly in patients who do not respond to standard approaches. Recently, botulinum toxin type A (BoNT-A) has been investigated as a novel therapeutic option for vasodilatory ETR [2]. Early studies indicate encouraging results, but the treatment has not yet been formally approved for this purpose. The objective of this review is to examine the available evidence on the effectiveness and safety of BoNT-A in managing rosacea.

## Review

Methodology

Study Design and Reporting Guidelines

In our review, we followed the PRISMA (Preferred Reporting Items of Systematic Reviews and Meta-Analyses) guidelines to ensure that studies were selected with the least amount of bias [3]. This study protocol was registered a priori in PROSPERO with the ID: CRD420251125238 [4]. Due to the nature of the study, ethical approval was not required.

Search Strategy

In August 2025, we conducted a systematic search of the PubMed (MEDLINE) and Google Scholar databases. Additional articles were identified from the bibliographies of reviewed articles. A search was conducted using the following keywords: (erythematotelangiectatic rosacea OR ETR OR facial redness OR rosacea subtype I) AND (botulinum toxin OR botulinum toxin type A OR BoNT-A OR onabotulinumtoxinA OR abobotulinumtoxinA). Based on PICOS (Population, Intervention, Comparator, Outcome, Study design) criteria, studies were considered for the review [5].

Eligibility Criteria

Inclusion criteria: For inclusion in our systematic review, the studies had to meet the following criteria: The population consisted of adult patients diagnosed with ETR based on clinical or standardized diagnostic criteria; the intervention was intradermal or microdroplet injections of BoNT-A; the comparator was placebo, no treatment, or standard treatment, such as topical metronidazole or laser therapy; the outcomes included clinical severity (redness/flushing scores), patient-reported outcomes, such as satisfaction or DLQI, biophysical measures, such as transepidermal water loss or erythema index and adverse events, and the study design was limited to randomized controlled trials (RCTs).

Exclusion criteria: Studies were excluded if the population included patients with non-erythematotelangiectatic subtypes of rosacea such as papulopustular or phymatous types or mixed subtypes, if the intervention used BoNT-A for non-rosacea indications or in combination without isolating its effects, if the comparator group or RCT design was absent such as in case series or cohort studies, if outcomes did not include clinical or biophysical outcomes specific to ETR, or if the study design was non-RCT, animal studies, case reports, conference abstracts, or review articles.

Study Selection Process

Two independent reviewers (RA and JS) screened papers simultaneously and independently by title and abstract using the Rayyan app for systematic reviews [6]. After that, the full texts of the articles were reviewed by the same two independent reviewers (RA and JS), with any differences resolved by a third reviewer (KM).

Data Extraction

Data extraction was performed by three reviewers (IA, MA, EA) for the following variables: total number of patients, number of patients in each group, outcomes being measured, age range, mean age in years with standard deviation, sex, inclusion and exclusion criteria, baseline rosacea severity, such as ETR grading by the Clinician’s Erythema Assessment (CEA) or IGA scale, duration of rosacea before treatment, type of BoNT-A used, including onabotulinumtoxinA, abobotulinumtoxinA, or incobotulinumtoxinA, dose, number of injection points, injection depth (intradermal vs. subdermal), technique used such as microinjection or mesotherapy, number of sessions, type of comparator group, such as placebo or topical therapy, primary efficacy outcomes, such as change in erythema score, assessment tools used, such as CEA, IGA, or patient satisfaction, time points of assessment, such as one week or four weeks, change in erythema score from baseline, reduction in telangiectasia, patient-reported outcomes, such as quality of life or satisfaction, adverse effects reported including details, randomization method, blinding of participants and assessors, incomplete outcome data, and selective reporting.

Risk-of-Bias Assessment

Studies included in the review were assessed for quality and risk of bias using the revised Cochrane risk-of-bias tool for randomized trials (ROB 2) [7]. The ROB-2 tool evaluates bias across five domains: bias arising from the randomization process, bias due to deviations from intended interventions, bias due to missing outcome data, bias in measurement of the outcome, and bias in selection of the reported result. All included studies were assessed independently by two reviewers (RA and JS), with any disagreements resolved by a third reviewer (KM).

Data Synthesis

A meta-analysis was initially planned to quantitatively synthesize the outcomes of the included studies. However, due to substantial clinical and methodological heterogeneity among the trials, a meta-analysis was not conducted. Instead, a qualitative synthesis of the study findings was performed.

Results

Study Selection

**Figure 1:**
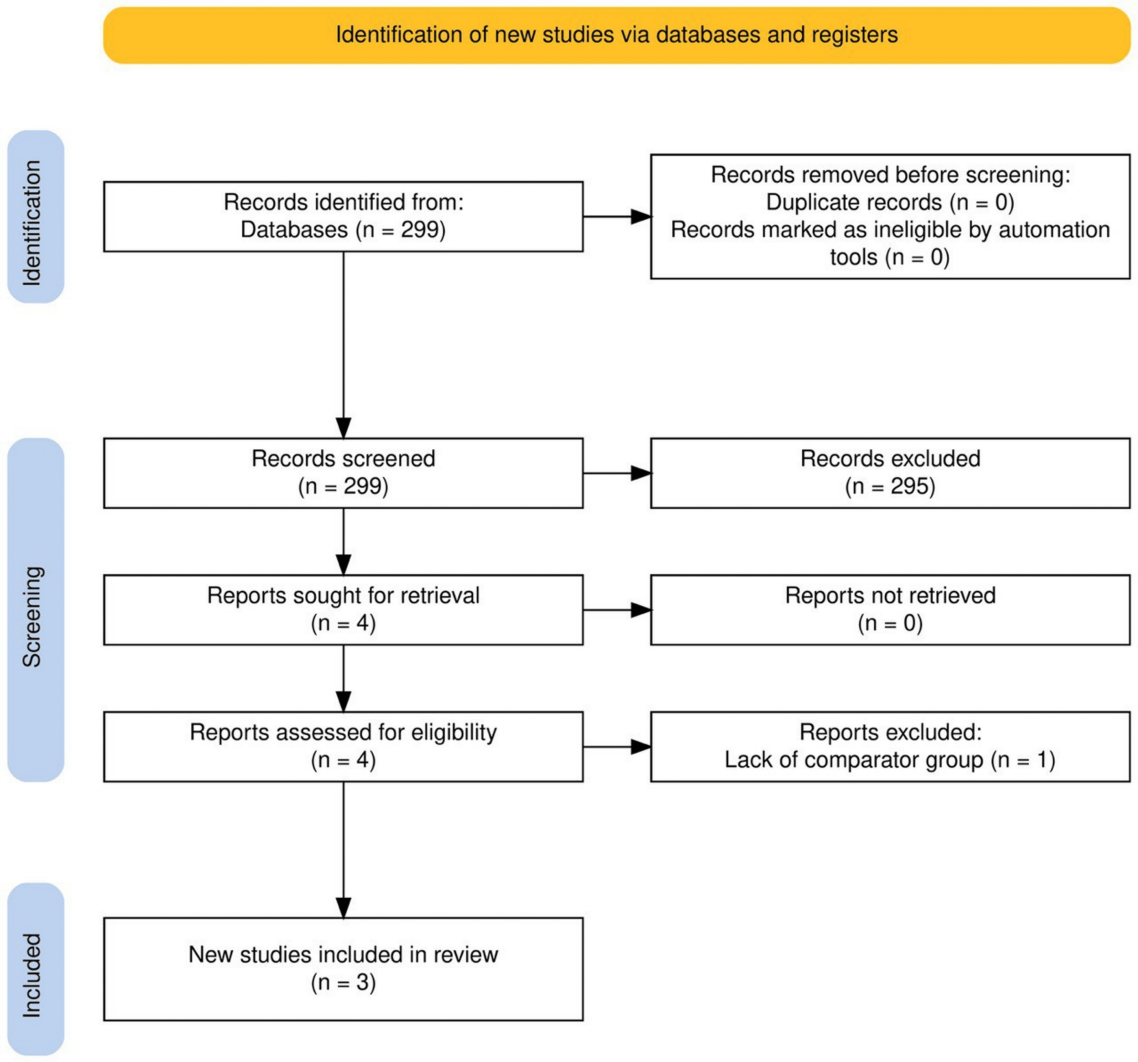
PRISMA flow diagram showing the selection of studies included in the systematic review, including records identified, screened, assessed for eligibility, and included, with reasons for exclusion at each stage PRISMA: Preferred Reporting Items of Systematic Reviews and Meta-Analyses

The systematic literature search identified a total of 299 publications, including 98 from PubMed and 201 from Google Scholar (first 20 pages). After removal of duplicates, 299 unique articles were screened by title and abstract, resulting in 4 full-text articles assessed for eligibility. Of these, three studies published between January 2017 and May 2025 met the inclusion criteria and were included in the qualitative synthesis (Figure 1).

Study Characteristics

All three studies were prospective, randomized, double-blind clinical trials. Two trials, conducted by Kim et al. (2019) and Dağtaş et al. (2025), employed a split-face design, with intradermal botulinum toxin injections administered to one side of the face and placebo (normal saline) to the contralateral side; both were single-center, randomized, double-blind, controlled studies [8,9]. In contrast, Dayan et al. (2017) conducted a parallel-group, randomized, double-blind, placebo-controlled pilot study in which participants received either intradermal incobotulinumtoxinA or saline [10]. The studies were performed in the United States, Korea, and Turkey [8-10].

Participant and Clinical Characteristics

A total of 62 adults were enrolled across 3 randomized controlled trials: Dayan et al. included nine participants, Kim et al. included 23 after one exclusion due to a protocol violation, and Dağtaş et al. recruited 30 participants [8-10]. All subjects were diagnosed with ETR; in comparison, Dayan et al. enrolled individuals presenting with both erythematotelangiectatic and papulopustular forms of rosacea [10]. Baseline disease severity in all studies was determined using validated assessment tools, including the CEA, the Rosacea Clinical Scorecard (RCS), and the Global Aesthetic Improvement Scale (GAIS). Participants’ ages ranged from 18 to 61 years, with an approximate mean age of 39.2 years. Sex distribution was reported in two studies, with 12 males (22.6%) and 41 females (77.4%) among 53 participants, indicating an expected predominance of female patients. Baseline comorbidities were not reported; however, all studies applied strict exclusion criteria to minimize confounding, excluding individuals with neuromuscular disorders (e.g., myasthenia gravis, Lambert-Eaton syndrome), autoimmune diseases (e.g., systemic lupus erythematosus), pregnancy or lactation, menopausal facial flushing, or recent aesthetic procedures or botulinum toxin treatments within the preceding six months. All interventions involved intradermal microinjections of BoNT-A. Dayan et al. administered 22 ± 3.4 injections of incobotulinumtoxinA (mean dose 17.1 ± 2.54 U) or saline placebo at baseline, with all participants receiving BoNT-A at week 16 (23 ± 5.0 injections, mean dose 17.3 ± 3.7 U) [10]. Kim et al. and Dağtaş et al. used a split-face design, administering BoNT-A (15 units prabotulinumtoxinA or 15 units onabotulinumtoxinA, respectively) to one side and normal saline to the other in a single session [8,9]. Efficacy was assessed with RCS and Subject Self-Satisfaction Evaluation (SSSE) (Dayan, weeks 1-20), CEA and GAIS (Kim, weeks 2-12), and CEA plus patient self-assessment (Dağtaş, 1 month), allowing direct comparisons of BoNT-A versus saline across formulations, doses, injection numbers, and time points [8-10]. Table 1 summarizes the technique and location of injection as well as other clinical characteristics of the patients included in the study.

**Table 1:** Summary of patient characteristics and procedural details across included studies, highlighting injection techniques, injection sites, and relevant demographic and clinical features for each study cohort. ETR: erythematotelangiectatic rosacea; PPR: papulopustular rosacea; BoNT-A: botulinum toxin type A; RCS: Rosacea Clinical Scorecard; SSSE: Subject Self-Satisfaction Evaluation; CEA: Clinician’s Erythema Assessment; GAIS: Global Aesthetic Improvement Scale

Study	N (analyzed)	Rosacea type	Age (years	Sex (M/F)	BoNT-A type & dose	Injection type & sessions	Comparator	Outcome measures	Assessment time points
Dayan et al. [10]	9	ETR or PPR	26-61 years	Not reported	IncobotulinumtoxinA 17.1 ± 2.54 U	Intradermal microinjections, avg. 22 ± 3.4 at baseline; all received BoNT-A at week 16, 23 ± 5.0 injections	Bacteriostatic saline	RCS, SSSE	Weeks 1, 4, 12, 16, 17, 20
Kim et al. [8]	23	ETR	21-49 years	6/17	PrabotulinumtoxinA (NABOTA) 15 U	Split-face intradermal microinjection, single session	Normal saline (other half of face)	CEA, GAIS	Weeks 2, 4, 8, 12
Dagtas et al. [9]	30	ETR	18-60 years	6/24	OnabotulinumtoxinA 15 U	Split-face intradermal microinjection, single session	Normal saline (other half of face)	CEA, Patient Self-Assessment	1 month

Clinical Outcomes, Patient-Reported Outcomes, and Safety

There was considerable heterogeneity in the assessment of patient satisfaction across the included studies, as more than three different evaluation tools were used; however, all studies consistently demonstrated high levels of patient satisfaction following intradermal BoNT-A treatment. Three prospective, randomized clinical trials evaluated the efficacy and safety of intradermal BoNT-A for ETR. Dayan et al. used the RCS and SSSE to assess outcomes at 1, 4, 12, 16, 17, and 20 weeks post-treatment, reporting a significant reduction in erythema scores from baseline at all time points (P < 0.05) and sustained improvement in redness and satisfaction without major adverse events [10]. Kim et al. employed the CEA and Global Aesthetic Improvement Scale (GAIS) at 2, 4, 8, and 12 weeks after treatment, demonstrating a significant reduction in erythema severity (P < 0.01) along with improvements in telangiectasia, patient-reported outcomes, quality of life, and overall satisfaction [8]. Reported complications included mild and self-limiting reactions, such as localized allergic responses, facial palsy, or transient paralysis around the injection sites. Dağtaş et al. assessed outcomes one month post-treatment using the CEA scale and patient self-assessment, reporting a significant reduction in erythema scores (P < 0.001) and improved satisfaction and quality of life, with only mild and transient adverse events, including localized tenderness, bruising, and one case of temporary facial muscle paresis [9]. A detailed summary of the included studies, assessment tools, outcomes, and reported complications is presented in Table 2. Overall, despite variability in both duration of study and assessment methods, all studies demonstrated statistically significant and clinically meaningful improvements in erythema severity and patient satisfaction following intradermal BoNT-A treatment, with a favorable safety profile and minimal, transient complications.

**Table 2:** Summary of included studies evaluating intradermal botulinum toxin (BTX) for erythematotelangiectatic rosacea

Study	Study design	Assessment tools	Time points of assessment	Main outcomes	Patient satisfaction	Reported complications
Dayan et al. [10]	Prospective, randomized, controlled, double-blind, parallel group study	Redness Clinical Score (RCS), Subject Self-Satisfaction Evaluation (SSSE)	1, 4, 12, 16, 17, 20 weeks post-treatment	Significant reduction in erythema score from baseline at all time points (P < 0.05)	Sustained improvement in satisfaction across all visits	None reported
Kim et al. [8]	Single-center, prospective, randomized, double-blind, split-face clinical study	Clinician Erythema Assessment (CEA), Global Aesthetic Improvement Scale (GAIS)	2, 4, 8, 12 weeks post-treatment	Significant reduction in erythema severity (P < 0.01); improvement in telangiectasia, quality of life, and overall satisfaction	High satisfaction reported	Mild, transient reactions including localized allergic responses, facial palsy, or temporary paralysis near injection sites
Dagtas et al. [9]	Prospective, randomized, controlled, double-blind, split-face study	Clinician Erythema Assessment (CEA), Patient Self-Assessment	1 month post-treatment	Significant reduction in erythema scores (P < 0.001); improved quality of life	High satisfaction reported	Mild tenderness, bruising, one case of transient facial muscle paresis

Risk-of-Bias Assessment

The risk-of-bias assessment, conducted using the Cochrane RoB 2.0 tool [7], indicated an overall low to moderate risk of bias among the included studies. The study by Dayan SH et al. demonstrated some concerns regarding the randomization process due to insufficient details on sequence generation and allocation concealment, as well as a very small sample size (n = 9) [10]. Additionally, there were concerns related to missing outcome data, with an attrition rate of approximately 22% and incomplete reporting on how missing data were handled. Although the study was described as randomized and double-blind, the absence of trial registration or a pre-published protocol introduced a potential risk of selective reporting. Overall, this study was judged to have some concerns for risk of bias. In contrast, both Kim et al. and Dağtaş et al. were assessed as low risk across most domains, including randomization, deviations from intended interventions, missing outcome data, and outcome measurement [8,9]. Minor concerns were noted for the absence of preregistration in Kim et al. [8], but this did not significantly impact the overall assessment. Consequently, the overall risk of bias for these two studies was considered low, while the study by Dayan et al. was rated as having some concerns [10]. A summary of the risk-of-bias evaluation for all included studies is presented in Table 3. However, the results should be interpreted with appropriate caution, as they are influenced by the study by Dayan et al., as studies with a higher risk of bias may overestimate treatment effects.

**Table 3:** Risk-of-bias assessment for the included randomized controlled trials using the Cochrane Risk of Bias 2.0 (RoB 2.0) tool

Study	Domain 1: bias arising from the randomization process	Domain 2: bias due to deviations from intended interventions	Domain 3: bias due to missing outcome data	Domain 4: bias in measurement of the outcome	Domain 5: bias in selection of the reported result	Overall assessment
Dayan et al. [10]	Some concerns: stated randomized, but no details on sequence generation or allocation concealment. Very small sample (n=9).	Low risk	Some concerns: extremely small sample size, 2 patients discontinued before completion (attrition ~22%). Missing data handling not fully described.	Low risk	Some concerns: no trial registration or pre-published protocol; selective reporting can’t be ruled out.	Some concerns (small n, poor randomization reporting, attrition).
Kim et al. [8]	Low risk	Low risk	Low risk	Low risk	Some concerns: no preregistration found; possibility of selective reporting.	Low risk
Dagtas et al. [9]	Low risk	Low risk	Low risk	Low risk	Low risk	Low risk

Discussion

Main Findings

This systematic review aimed to evaluate the current evidence regarding the efficacy and safety of intradermal BoNT-A in the management of ETR [11-13]. Three randomized controlled trials involving a total of 62 adult participants met the inclusion criteria [11-13]. Across all studies, BoNT-A demonstrated significant reductions in facial erythema and flushing severity, as measured by validated clinical scales such as the CEA, RCS, and GAIS [11,12]. Improvements were consistently accompanied by high levels of patient satisfaction and enhanced quality of life [11,13]. Adverse events were mild, transient, and self-limiting, including temporary localized reactions and rare transient muscle weakness [11,13]. Despite heterogeneity in study design, injection technique, and assessment tools, the results collectively indicate that BoNT-A is a promising and well-tolerated therapeutic option for patients with vasodilatory ETR who are unresponsive to conventional treatments [12,14].

Comparison With Existing Literature

All included studies demonstrated that intradermal BoNT-A led to significant reductions in facial erythema and flushing severity, with improvements observed on validated scales such as the CEA, RCS, and GAIS [11,12]. These results are consistent with earlier observational and pilot studies suggesting that BoNT-A can reduce cutaneous vasodilation by inhibiting acetylcholine and vasoactive neuropeptide release from peripheral nerve endings [13,14]. Additionally, all included trials reported high levels of patient satisfaction and improved quality of life following treatment [11,13]. Reported adverse effects were mild and transient, including localized bruising, tenderness, or temporary facial weakness, aligning with previously reported safety profiles in aesthetic and dermatologic applications of BoNT-A [11,14].

Collectively, the evidence suggests that BoNT-A represents a promising, well-tolerated, and minimally invasive option for managing persistent erythema in ETR, particularly for patients who are refractory to conventional therapies such as topical α-adrenergic agonists, azelaic acid, or pulsed-dye laser treatments [12,14]. However, the small sample sizes and methodological heterogeneity among current RCTs highlight the need for larger, multi-center trials with standardized protocols to confirm the therapeutic role of BoNT-A in rosacea management [12,14].

Implications for Clinical Practice

The findings of this systematic review suggest that intradermal BoNT-A may serve as a valuable adjunctive or alternative therapy for patients with ETR who do not achieve satisfactory outcomes with conventional treatments such as topical α-adrenergic agonists, azelaic acid, oral doxycycline, or laser therapy [12,14]. The consistent reduction in erythema severity and improvement in patient satisfaction observed across the included randomized controlled trials indicate that BoNT-A could play a meaningful role in addressing the neurovascular and neurogenic inflammation underlying persistent facial flushing [13,14]. Clinically, BoNT-A offers a minimally invasive, office-based procedure with a favorable safety profile and relatively prolonged therapeutic duration of up to three to five months, potentially improving adherence and patient quality of life [11,13]. Moreover, the transient and mild nature of reported adverse effects supports its feasibility in dermatologic practice [11,14].

However, given the limited number of randomized trials and variations in dosing regimens, injection techniques, and follow-up durations, BoNT-A should currently be considered an off-label intervention to be used cautiously in selected patients who are refractory to first-line therapies [12,14]. Clinicians should ensure appropriate patient counseling, informed consent, and careful intradermal injection technique to minimize complications such as localized weakness or asymmetry [11,13]. Future incorporation of BoNT-A into standardized rosacea management algorithms will depend on the outcomes of larger, multicenter, placebo-controlled studies that establish optimal dosing, treatment intervals, and long-term safety [12,14].

Comparison With Current Standard of Care

Current clinical guidelines for ETR recommend topical α-adrenergic agonists, such as brimonidine 0.33% gel and oxymetazoline 1% cream, as first-line therapies for persistent facial erythema, owing to their ability to induce transient vasoconstriction through selective α2- and α1A-adrenergic receptor stimulation [12,14]. These agents typically produce visible erythema reduction within 30 to 60 minutes of application, with effects lasting up to 12 hours; however, their benefits are temporary and often accompanied by rebound erythema or irritation upon discontinuation [11]. Laser and light-based therapies, including pulsed-dye laser (PDL) and intense pulsed light (IPL), are also well-established treatment modalities that target hemoglobin to thermally ablate telangiectatic vessels, offering longer-lasting improvement but requiring multiple sessions, specialized equipment, and careful patient selection to minimize adverse effects such as post-inflammatory hyperpigmentation and purpura [12,14].

In contrast, BoNT-A provides a novel, mechanism-based approach by modulating neurovascular signaling and inflammatory mediators, rather than relying solely on vascular constriction or photothermal destruction [13,14]. By inhibiting acetylcholine and vasoactive neuropeptide release from peripheral nerve endings, BoNT-A reduces vasodilation and neurogenic inflammation, leading to sustained improvement in erythema and flushing over several months [13,14]. Unlike topical vasoconstrictors, BoNT-A does not induce rebound redness, and its duration of effect (approximately 3-5 months) is comparable to or longer than that of light-based therapies, with minimal downtime and a favorable safety profile [11,13]. Nevertheless, BoNT-A remains off-label for rosacea, and optimal injection protocols have yet to be standardized [12,14]. Therefore, while it cannot yet replace first-line therapies, BoNT-A may serve as an effective adjunct or second-line option for patients who experience inadequate response or intolerance to conventional topical or device-based treatments [12,14].

Limitations

This review has several limitations. Only 3 RCTs met the inclusion criteria, with a total of 62 participants, limiting statistical power and generalizability [11-13]. Considerable clinical and methodological heterogeneity existed, including differences in study design (split-face vs. parallel-group), BoNT-A formulations, dosing regimens, injection points, follow-up durations, and assessment time points [11-14]. Outcome measures were not standardized; erythema severity and patient satisfaction were assessed using different tools, making direct comparisons and quantitative synthesis challenging [12,13]. Some studies lacked trial preregistration, detailed randomization procedures, or clear allocation concealment, introducing potential bias [12]. Follow-up periods were short (<20 weeks), and most studies were single-center with predominantly female participants, limiting external validity [12-14]. Due to heterogeneity in study designs, interventions, and outcomes, a meta-analysis was not possible, and only qualitative synthesis could be performed, restricting the ability to draw reliable conclusions [12].

Recommendations for Future Research

Future research should address existing gaps by conducting larger, multi-center randomized controlled trials that include diverse populations, standardized intervention protocols, and longer follow-up periods to assess the sustained efficacy and safety of BoNT-A [12,14]. Researchers should adhere to established reporting guidelines, such as CONSORT for clinical trials and PRISMA for systematic reviews, to ensure transparency, reproducibility, and methodological rigor [12].

Standardization of outcome measures for erythema severity, flushing frequency, and patient satisfaction is essential to enable meaningful comparisons and facilitate quantitative synthesis [12,13]. Future systematic reviews and meta-analyses should perform sensitivity and subgroup analyses to assess potential biases and explore heterogeneity, including rosacea subtype, toxin formulation, or injection technique [12,14]. Where feasible, individual patient data (IPD) meta-analyses could improve precision, adjust for confounders, and clarify patient-level predictors of response [12,14].

## Conclusions

The effectiveness and safety of intradermal botulinum toxin type A (BoNT-A) in the treatment of erythematotelangiectatic rosacea (ETR) are assessed in this systematic study. Based on specific clinical measures, the analysis of 3 randomized controlled trials with 62 adult patients shows that BoNT-A considerably decreases the severity of facial erythema and flushing. According to the results, adverse effects were minor and temporary, and patient satisfaction and quality of life were notably improved.

Given the encouraging results, larger, comprehensive trials must be conducted to validate them due to the small number of studies and variability in the current literature at hand. The main goals of future studies should aim for standardizing treatment procedures and evaluating long-term safety and efficacy. Considering its good safety record and long half-life, adding BoNT-A to clinical practice as therapy for patients who don't respond to traditional treatments is a promising alternative. BoNT-A exhibits great potential, but more research is necessary to determine its place in the overall treatment of rosacea.
